# Evaluating the dosimetric effect of treatment-induced changes in virally mediated head and neck cancer patients

**DOI:** 10.1002/jmrs.30

**Published:** 2013-11-20

**Authors:** Elizabeth Brown, Rebecca Owen, Kerrie Mengersen, Fiona Harden, Sandro Porceddu

**Affiliations:** 1 Radiation Oncology Department, Princess Alexandra HospitalBrisbane, Queensland, Australia; 2 Queensland University of TechnologyBrisbane, Queensland, Australia; 3 Radiation Oncology Department, Radiation Oncology Mater CentreBrisbane, Queensland, Australia; 4 School of Medicine, University of QueenslandBrisbane, Queensland, Australia

**Keywords:** Head and neck cancer, HPV-16, planning, radiation therapy

## Abstract

**Introduction:**

Patients with virally mediated head and neck cancer (VMHNC) often present with advanced nodal disease that is highly radioresponsive as demonstrated by tumour and nodal regression during treatment. The resultant changes may impact on the planned dose distribution and so adversely affect the therapeutic ratio. The aim of this study was to evaluate the dosimetric effect of treatment-induced anatomical changes in VMHNC patients who had undergone a replan.

**Methods:**

Thirteen patients with virally mediated oropharyngeal or nasopharyngeal cancer who presented for definitive radiotherapy between 2005 and 2010 and who had a replan generated were investigated. The dosimetric effect of anatomical changes was quantified by comparing dose–volume histograms (DVH) of primary and nodal gross target volumes and organs at risk (OAR), including spinal cord and parotid glands, from the original plan and a comparison plan.

**Results:**

Eleven three-dimensional conformal radiation therapy (3DCRT) and two intensity modulated radiation therapy (IMRT) plans were evaluated. Dose to the spinal cord and brainstem increased by 4.1% and 2.6%, respectively. Mean dose to the parotid glands also increased by 3.5%. In contrast, the dose received by 98% of the primary and nodal gross tumour volumes decreased by 0.15% and 0.3%, respectively, when comparing the initial treatment plan to the comparison plan.

**Conclusion:**

In this study, treatment-induced anatomical changes had the greatest impact on OAR dose with negligible effect on the dose to nodal gross tumour volumes. In the era of IMRT, accounting for treatment-induced anatomical changes is important as focus is placed on minimizing the acute and long-term side effects of treatment.

## Introduction

The emergence of virally mediated head and neck cancers (VMHNC) has presented the oncology community, and in particular radiation oncology, with some unique challenges. These patients generally present younger, in better health compared with historical head and neck cancer patients, have radioresponsive disease and good prognosis, meaning that the development of strategies to minimize their long-term side effects is vital.^1^

Head and neck cancer patients often experience numerous anatomical changes during treatment.^2^ These can be externally visible, including tumour and nodal regression and weight loss, and internal, including parotid gland volume changes.^3^,^4^ These changes may result in differences in dose distribution, causing potential underdosing of target volumes and/or overdosing of surrounding normal and critical tissue.^5^ This is of particular importance when highly conformal techniques, such as intensity modulated radiation therapy (IMRT) techniques, are used because of the steep dose gradients that can be created between target volumes and surrounding normal and critical tissues.^6^ Adaptive radiotherapy is one dosimetric approach that can be employed to account for ongoing treatment-induced changes in anatomy and so minimize the impact on highly conformal IMRT dose distributions.^7^

This study was a retrospective review of patients with VMHNC, who attended the Princess Alexandra Hospital (PAH) for definitive radiotherapy between 2005 and 2010. Patients who underwent a replan, due to anatomical changes identified by radiation therapists during their radiotherapy treatment course, were investigated. Both virally mediated nasopharyngeal cancer (NPC) and oropharyngeal cancer (OSCC) were studied, as collectively these VMHNCs represent a subset of cancers that are clinically distinct.^8^ They have a greater likelihood of response to therapy, are not necessarily related to smoking, have a more favourable prognosis and follow a different pathway of malignant transformation.^8^ The primary aim of this investigation was to evaluate the effect of treatment-induced anatomical changes, such as weight loss and tumour or nodal shrinkage, on the planned dose distribution to assist in the development of appropriate adaptive radiotherapy strategies.

## Methods and Materials

### Patients

Patients with VMHNC who received definitive radiotherapy treatment with or without systemic therapy, between 2005 and 2010, were identified from a prospective head and neck database at PAH. Eligibility criteria included histologically confirmed NPC or OSCC, with either positive serology for Epstein–Barr virus (EBV) or human papillomavirus (HPV) (p16 immunostaining >70%), respectively, and node-positive disease with any T-stage disease and treatment plan accessible on the treatment planning system. Patients who had a replan generated during their treatment were selected in order to examine the volumetric and dosimetric changes between the planning scan and the repeat planning computed tomography scan (re-CT). At the time of the study, there was no protocol to identify patients requiring replanning and decisions were made based on the treating radiation therapists' judgement on a daily basis. These decisions were informed by the evaluation of the mask fit and assessment of weekly separation measurements. If a change in separation reading of greater than 1 cm occurred, the plan was returned to planning for review and potential re-CT. Patient demographics and tumour characteristics, including pretreatment size of the dominant node, were recorded. Nodal size data were collected from each patient's diagnosis and staging information. Patient weight was measured by a radiation oncology nurse or dietician at the time of planning and at re-CT. The project was approved by the Human Research Ethics Committees from the PAH and Queensland University of Technology.

### Re-CT and volumetric change evaluation

Each patient's re-CT was performed in the same position as the planning CT. The re-CT was manually fused with the planning CT using the registration match point/region prescribed by the radiation oncologist and this registration was checked by both a senior radiation therapist and the radiation oncologist. The primary and nodal gross target volumes (GTV-p and GTV-n) and specific organs at risk (OAR) were recontoured by a radiation oncologist on the re-CT to determine if any volumetric changes had occurred. The same radiation oncologist did not contour these volumes on both the planning CT and re-CT in all cases. The volumes were recorded for the GTV-p and GTV-n and left and right parotid glands.

### Dosimetric effect evaluation

The effect of any treatment-induced anatomical changes on the dose distribution was quantified by comparing the primary plan with a comparison plan. All plans were calculated on the Eclipse treatment planning system (version 8.6; Varian Medical Systems, Palo Alto, CA). The primary plan was calculated using the original CT data. The comparison plan used the same treatment fields from the primary plan, but was calculated on the re-CT data. The monitor units (MU) for all treatment fields remained the same for both plans to ensure that the dosimetric effect of anatomical changes could be accurately recorded. For three-dimensional conformal radiation therapy (3DCRT) plans, the comparison plan was created by one radiation therapist who replicated the primary plan, at the same isocentre position, using the re-CT data. The plan was then calculated and doses adjusted to represent the treatment portions delivered before and after the observation of anatomical changes. For IMRT plans the fluence map from the primary plan was used to calculate the comparison plan. The dosimetric effect was quantified by comparing dose–volume histograms (DVH) of GTV-p and GTV-n and OAR from both plans. OAR investigated were spinal cord, brainstem, and left and right parotid glands.

### Statistics

Data were analysed using the Stata (version 12.1; StataCorp LP, College Station, TX) program. Doses to target volumes and OAR were recorded from both the initial and comparison plans. The re-CT data were also examined to assess volumetric changes in tumour, nodal and parotid volumes, and weight loss. Statistical analysis included basic descriptive statistics to determine the impact of treatment-induced anatomical changes on the dose distribution. Mann–Whitney and Wilcoxon tests were used to analyse the comparison of changes in volume and dose of target volumes and OAR for the initial plan and the comparison plan. A *P*-value of 0.05 was considered statistically significant.

## Results

### Patient characteristics

Sixteen patients had a replan calculated and were selected for the study. Three patients were excluded from this investigation. Of these, one was unable to be retrieved from archive, one primary treatment plan was not utilized as the patient returned at a later date to receive treatment, and one was only planned to receive a dose of 50 Gy. The demographics of these patients are given in Table 1. Eleven patients were male and two were female, ten patients had HPV-positive OSCC and three had EBV-positive NPC. Five (50%) of the oropharynx patients were staged as having T2-3N2 disease and two (66.7%) nasopharynx patients, T4N2 disease. Eleven patients in this study were treated with 3DCRT and two patients were treated with IMRT. All patients were prescribed and treated to a total dose of 70 Gy in 35 fractions. The details of the prescription and OAR tolerances used are outlined in Table 2. At least one parotid gland was spared where possible, ideally to a mean of <26 Gy, but up to a mean of 33 Gy, as the specified tolerance dose.^9^ Replans were generated at a mean time point of fraction 22 (range 17–29). Eight (61.5%) patients underwent a re-CT and replan due to a combination of two factors: weight loss and tumour and/or nodal regression. The reason for re-CT and replan for the remaining five patients were weight loss or tumour regression alone and a prescheduled replan of the lower neck area. Only 3 of the 13 (23.1%) patients actually had the plan from the re-CT clinically implemented. This was due to the fact that the DVHs of target and OAR were considered clinically unacceptable by the treating radiation oncologist.

**Table 1:** Patient characteristics.

Characteristics	
Sex	
Male	11
Female	2
Age mean (range)	50 (36–64)
Primary tumour site	
Tonsil	6
Base of tongue	4
Nasopharynx	3
T-classification	
1	1
2	5
3	3
4	4
N-classification	
1	1
2	8
3	4
Smoking history	
Never	4
Former	3
Active	6
Nodal size mean (range)	48.8 mm (24–90 mm)
Treatment technique	
3DCRT	11
IMRT	2
Mean timing of re-CT	#22 (#17–#29)
Replan implemented	3

3DCRT, three-dimensional conformal radiation therapy; IMRT, intensity modulated radiation therapy; CT, computed tomography.

**Table 2:** Prescription and organ at risk dose tolerances.

Structure	Dose
Prescription	70 Gy in 35#
Spinal cord	≤45 Gy
Brainstem	≤54 Gy
Parotid glands	Mean dose ≤33 Gy

### Weight loss and volume reduction

**Figure 1:**
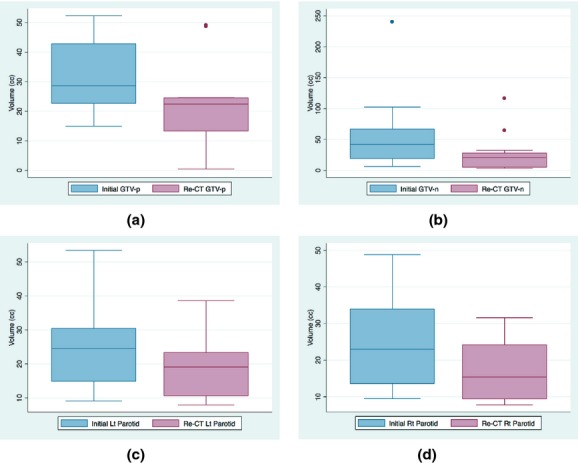
Volume changes in (a) primary gross target volume (GTV-p), (b) nodal gross target volume (GTV-n), (c) left parotid gland, and (d) right parotid gland between the planning CT and Re-CT. The box represents standard deviation, and the horizontal line in the box represents the mean of the volumes. The bar represents the range of the volumes. The dots represent outlying measurements.

The weight loss and volume changes for GTVs and the parotid glands are demonstrated in Figure 1 and Table 3. All patients experienced weight loss during treatment. The overall mean percentage weight loss was 6.5%. Reduction in volume was observed for both target volumes and all OAR studied; however, only the GTV-p and GTV-n approached statistical significance (*P* = 0.06 and *P* = 0.09). The greatest mean volume reduction seen was for the GTV-n with a 50.8% reduction being recorded. One patient was excluded from the GTV-p results due to incomplete volumes. The greatest percentage mean volume change for OAR was recorded for the parotid glands with a volume reduction of 21.8% and 26.4% for the left and right glands, respectively.

**Table 3:** Mean weight and volume reduction details.

Structure	Planning CT (range)	Re-CT (range)	Difference	*P*-value
Mean weight (kg)	81.7 (51.8–127.5)	76.4 (42.9–116.6)	5.3 (6.5%)	0.40
Mean GTV-p^1^ volume (cc)	32.3 (14.9–52.3)	22.6 (0.4–49.1)	9.7 (30%)	0.06
Mean GTV-n volume (cc)	56.1 (6.4–240.2)	27.6 (3.8–116.6)	28.5 (50.8%)	0.09
Mean left parotid volume (cc)	24.8 (9.1–53.4)	19.4 (7.9–38.6)	5.4 (21.8%)	0.27
Mean right parotid volume (cc)	25.0 (9.5–48.8)	18.4 (7.8–31.6)	6.6 (26.4%)	0.15

GTV-p, primary gross target volume; GTV-n, nodal gross target volume; CT, computed tomography.

* One patient excluded due to incomplete voluming.

### Dosimetric effect

The details of the dose comparison between the primary plan and the comparison plan are demonstrated in Table 4. The mean dose encompassing 98% of the GTV-p and GTV-n volumes (D98) was slightly decreased when comparing the primary plan with the comparison plan. In contrast, the mean doses to all OAR investigated increased with the greatest increase being for the maximum spinal cord dose (4.1%). Eleven (84.6%) patients had an increase in dose to two or more OAR when comparing the primary plan with the comparison plan. While none of the differences was statistically significant (*P* > 0.05), greater dose variations and larger standard deviations were observed for the OAR in comparison to the target volumes. The GTV-p had a range of 61.7–70.1 Gy and the GTV-n had a range of 58.9–71.1 Gy. The left parotid had a range of 35.1–69.3 Gy and the right parotid had a range of 31.3–68.6 Gy. This is consistent with the primary planning objective of covering the target volumes with the prescribed dose.

**Table 4:** Mean doses to tumour and organ at risk volumes.

Structure	Primary plan ± SD (Gy)	Comparison plan ± SD (Gy)	Difference (Gy)	*P*-value
Mean GTV-p D98*	66.9 ± 2.6	66.8 ± 2.7	−0.1 (−0.15%)	0.95
Mean GTV-n D98	67.6 ± 2.8	67.4 ± 3.9	−0.2 (−0.3%)	0.83
Mean plan max	77.2 ± 1.8	76.9 ± 1.9	−0.3 (−0.39%)	1.00
Mean spinal cord max	43.6 ± 3.8	45.4 ± 4.8	1.8 (4.1%)	0.47
Mean brainstem max	42.1 ± 14.0	43.2 ± 11.8	1.1 (2.6%)	0.88
Left parotid mean	51.9 ± 12.6	53.6 ± 12.0	1.7 (3.3%)	0.50
Right parotid mean	45.0 ± 13.1	46.6 ± 14.3	1.6 (3.6%)	0.80

SD, standard deviation; GTV-p, primary gross target volume; GTV-n, nodal gross target volume.

* One patient excluded due to incomplete voluming.

The observed range of treatment-induced anatomical changes experienced resulted in a larger dosimetric effect in some patients. In one patient, anatomical changes resulted in only a minimal change in target volume D98 dose (−0.2 and 2.2% in GTV-p and GTV-n, respectively), but a much greater impact on OAR doses; 4.2% increase in maximum spinal cord dose and 13.4% and 8% increase in left and right parotid gland mean doses, respectively.

## Discussion

This study showed that treatment-induced anatomical changes had the greatest impact on the OAR, increasing the doses received with negligible dose decrease to the primary and nodal GTVs. These results, while not statistically significant, are of clinical importance as the observed dosimetric impact could result in the tolerance dose of an OAR being exceeded. As an example, one patient studied was originally planned to receive a maximum spinal cord dose of 46.2 Gy, but due to treatment-induced anatomical changes received 54.1 Gy. This overdosing of OAR can result in increased acute and long-term toxicity experienced by the patient and a reduction in their overall quality of life. Consequently, it is imperative that the effect of these anatomical changes is considered and accounted for. This is of particular importance with the parotid glands as, due to their steep dose–response relationship, exceeding the tolerance dose could result in permanent loss of salivary function.^10^

Previous studies have also demonstrated the decrease in volume and subsequent increase in dose received by the parotid glands.^7^,^11^–^14^ Beltran and colleagues^15^ have reported an increase of 2.5% in spinal cord dose and an increase of 4.7% and 6.7% in mean parotid gland dose during head and neck IMRT. In contrast to this study, they also reported a significant decrease in dose (D98) to the primary target volumes (*P* = 0.01).^15^ This difference could be due in part to the fact that only GTV dose coverage was measured in this study, whereas the study by Beltran primarily reported the planning target volume (PTV) dose. When specifically focussing on the GTV D98 dose, only a slight effect is observed: a 0.2% decrease in this study and a 0.4% increase in the Beltran study. In addition, only patients receiving IMRT were reported by Beltran and colleagues, whereas the majority of patients in this study were treated with 3DCRT. It is well recognized that the steep dose gradients created with IMRT can make it more sensitive to treatment-induced anatomical changes, resulting in potential underdosing and/or overdosing of target and OAR volumes.^5^,^12^,^15^ The dosimetric effect observed in patients who received 3DCRT may be magnified under IMRT, particularly for OAR such as the parotid glands which are known to shift medially into higher dose areas with weight loss and tumour regression.^3^ The added workload associated with the replanning process can place substantial burden on busy radiotherapy departments and further highlights the importance of appropriate evidence-based adaptive radiotherapy protocols for head and neck IMRT.^16^ The wide range of parotid gland doses observed in the study may be related to a number of factors: initial parotid size, differences in disease pathology, differential shrinkage of the surrounding nodes and the improvement in radiotherapy treatment techniques used over the study period.

The adaptive radiotherapy protocols developed as part of this study will be tested in conjunction with daily image guidance using volumetric imaging allowing the exact timing of treatment-induced anatomical changes to be determined. This will enable accurate evaluation of their dosimetric impact and will maximize the benefit of adaptive intervention. Comprehensive assessment of the dosimetric impact of anatomical changes was not possible in this study as daily volumetric imaging was not used. As a result, the exact time point at which they occurred could not be determined. The re-CTs used for dosimetric evaluation were performed after treatment staff had observed the anatomical changes.

The inherent risk in retrospective studies, particularly the lack of control over data consistency, in combination with the small sample size means that cautious interpretation of the results should be undertaken. It is likely that the small sample size meant the study was insufficiently powered to detect any significant differences. Another limitation of this study was that the variability in contouring of target and OAR volumes between the planning CT and re-CT was not accounted for and may have impacted the volumetric and dosimetric results. Nonetheless, the results of this study support the dosimetric impact of treatment-induced changes and provide baseline data for the development of appropriately focussed adaptive treatment strategies. The clinically important dosimetric effect on OAR demonstrated in this study warrants further investigation with a larger sample size.

## Conclusion

In this study, treatment-induced anatomical changes had the greatest impact on OAR dose. The dosimetric impact observed is of clinical consequence and could potentially lead to exceeding an OAR tolerance dose. The development of adaptive radiotherapy strategies targeted at reducing OAR dose, while maintaining target volume dose, will be of great importance to patients' long-term quality of life and departmental efficiency. In the era of IMRT, accounting for treatment-induced anatomical changes is paramount due to the steep dose gradients between target volumes and OAR. This is of particular relevance to patients with VMHNC as their increased responsiveness to radiotherapy often leads to a favourable prognosis. Although this study investigated only VMHNC, the adaptive radiotherapy strategies developed using these findings and associated dosimetric impact will be evaluated in a larger prospective study, including all head and neck cancer patients with stratification between VMHNC and non-VMHNC patients.
